# Preparation, Structure and Properties of Epoxy/Carbonyl Iron Powder Wave-Absorbing Foam for Electromagnetic Shielding

**DOI:** 10.3390/polym16050698

**Published:** 2024-03-04

**Authors:** Xiaoli Liu, Hao Huang, Haijun Lu

**Affiliations:** 1 Composite Technology Center, AVIC Beijing Aeronautical Manufacturing Technology Research Institute, Beijing 101300, China; lxl_nanliu@163.com (X.L.); 15652919114@163.com (H.H.); 2 National Key Laboratory of Advanced Composites, AECC Beijing Institute of Aeronautical Materials, Beijing 100095, China

**Keywords:** carbonyl iron powder, absorber, foam, reflectivity

## Abstract

The application of absorbing materials for electromagnetic shielding is becoming extensive, and the use of absorbents is one of the most important points of preparing absorbing foam materials. In this work, epoxy resin was used as the matrix and carbonyl iron powder (CIP) was used as the absorbent, and the structural absorbing foam materials were prepared by the ball mill dispersion method. Scanning electron microscopy showed that the CIP was evenly dispersed in the resin matrix. The foam structures formed at pre-polymerization times of 10 min, 30 min and 50 min were analyzed, and it was found that the cell diameter decreased from 0.47 mm to 0.31 mm with the increase in the pre-polymerization time. The reflectivity of the frontal and reverse sides of the foam gradually tends to be unified at frequencies of 2–18 GHz. When the CIP content increased from 30 wt% to 70 wt%, the cell diameter increased from 0.32 mm to 0.4 mm, and the uniformity of CIP distribution deteriorated. However, with the increase in the CIP content, the absorption properties of the composite materials were enhanced, and the absorption frequency band broadened. When the CIP content reached 70 wt%, the compression strength and modulus of the foam increased to 1.32 MPa and 139.0 MPa, respectively, indicating a strong ability to resist deformation.

## 1. Introduction

**Figure 1:**
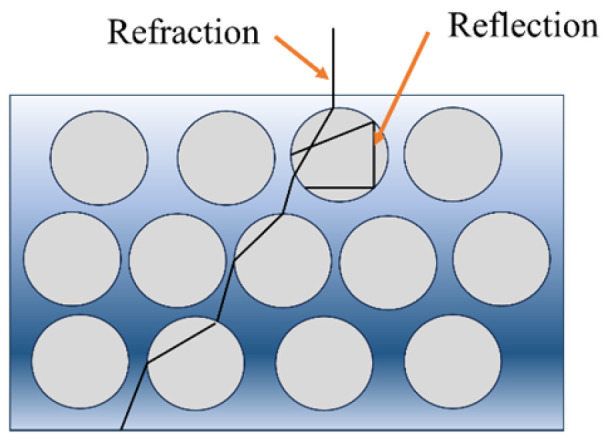
Microwave transport path in porous material.

The widespread use of electromagnetic technology has brought convenience to life, but the harm caused by electromagnetic radiation has gradually attracted attention [1,2]. Absorbers can absorb or shield electromagnetic waves, thereby controlling electromagnetic wave radiation within a specified range. Structural absorption foam is a structural–functional material with excellent electromagnetic wave absorption properties, a high specific strength, a light weight and features such as sound absorption and shock absorption. It is suitable for the manufacturing and processing of components with complex shapes and full-height sandwich structures; thus, absorbing materials have also become an important research topic in the military [3,4] such as achieving microwave stealth. In structural absorbing foams [5], microwaves propagate between the pores and the absorbing substrate through reflection and refraction [6,7]. The distance is longer than when propagating in a straight line of homogeneous materials, and the loss of microwaves is increased. Meanwhile, the microwaves entering the aperture are reflected many times and absorbed by the absorption matrix of the aperture wall, as shown in Figure 1. The transmission distance and loss of microwaves in the material are increased through the porous absorbing structure, which has also achieved greater reflection attenuation than sheet structure materials [8].

Generally speaking, both structural absorbers and coated absorbers need to add absorbers that absorb electromagnetic waves. The performance of absorbers often has a decisive effect on the absorption effect of materials. Therefore, absorbents are the core of microwave absorption technology. Compared to conventional absorbers, high-temperature absorbers are open-operated at a temperature of up to 300 °C or more [9,10].

Absorbents [11] are divided into dielectric loss types and magnetic loss types according to their principle. Dielectric loss absorbers mainly absorb electromagnetic waves through the interaction with the electric field [12]. The absorption efficiency depends on the dielectric constant of the material. This type of absorbers mainly includes resistive absorbers represented by carbon black, silicon carbide, special carbon fibers and dielectric absorbers represented by barium titanate ferroelectric ceramics.

The attenuation of electromagnetic waves by magnetic loss absorbers mainly comes from magnetic losses, such as ferrite [13] and carbonyl iron powder (CIP) [14,15]. CIP is a magnetic loss absorber with good absorbing properties. Due to the advantages of high microwave permeability, small matching thickness, high-temperature stability and high saturation magnetization, CIP has a wide range of application prospects and development potential in the field of stealth technology [16]. Folgueras et al. [15] prepared two different paint formulations containing carbonyl iron and/or polyaniline using polyurethane as a matrix. The carbonyl iron in the paints contributed to the dissipation of electromagnetic energy due to magnetic anisotropy effects, a characteristic of this material for frequencies larger than 2 GHz. Zhou et al. [17] prepared microwave-absorbing composites with thin thicknesses and wideband absorption through a spraying method using carbonyl iron particles as absorbers and silicone resin as the matrix. A value of reflection loss below −5 dB can be obtained in the frequency range of 5.76–18 GHz for a composite with an 0.8 mm thickness. Song et al. [18] prepared polymer composites based on a toughened polymer with various mass ratios of flake carbonyl iron powders (FCIs). The prepared toughened polymer obviously increased the permittivity for a high FCI content. Wang et al. [19] prepared SiO_2_-coated carbonyl iron particles using the Stober process and composites which use polyimide resin as a matrix. When the content of SiO_2_-coated carbonyl iron is 60 wt%, the value of minimum reflection loss decreases from −25 dB to −33 dB as the thickness increases from 1.5 mm to 2.1 mm. Yang et al. [20] fabricated a magnetic–dielectric composite of carbonyl iron/carbon foam with a three-dimensional reticulated structure by carbonization and vacuum impregnation. This structure provides a well-defined conductive path and controllable conductivity–temperature characteristics for optimizing dielectric losses as well as delaying the attenuation of magnetic losses at high temperatures. Mrlík et al. [21] prepared a conducting polymer, namely poly(2-(1H-pyrrole-1-yl)ethyl methacrylate, and obtained a substantial conducting shell on the magnetic core when grafted from the surface of carbonyl iron. Shen et al. [22] confirmed that CIP–calcium particles with core–shell structures formed during the hydration of the composites without fly ash. A core–shell structure can suppress the skin effect and the dielectric loss of the cementitious composites, enhancing their impedance matching and microwave absorption. Huang et al. [23] designed surface-shape-dependent CIP absorbers via surface coating with zinc oxide (ZnO) nanoparticles and a thermal annealing treatment. Samples of CIP@ZnO annealed at 350 °C, which have cubic cone ZnO nanoparticles, exhibit a minimum reflection loss of −55.35 dB at a thickness of 2.1 mm and a maximum effective absorption bandwidth of 7.09 GHz at a thickness of 2.0 mm.

To achieve the effect of electromagnetic shielding in a high-temperature environment, the foam material needs to have good stability. Epoxy resin is a commonly used organic material with good thermal stability [24,25,26], so it is used as a matrix. Furthermore, filler dispersion is very important for filler-filled polymer composites [27,28,29,30], and dispersion methods usually affect the filler dispersion. Therefore, in this work, a wave-absorbing foam material composed of epoxy resin [31] and CIP was first prepared by the hot melt in situ stirring method and the ball milling dispersion method. The particle morphology of the two preparation methods was compared using a scanning electron microscopy test. Then, characterization tests of the electromagnetic parameters, foam reflectivity and compressive strength were carried out. The effects of the pre-polymerization time and CIP content on the cell morphology, absorption properties, compression strength and modulus of foam were analyzed.

## 2. Experimental

### 2.1. Materials

Epoxy resin (bisphenol A type, E-51, epoxy value of 0.51 mmol/100 g) was produced by Nantong Xingchen Synthetic Materials Co., Ltd., Nantong, China. Diaminodiphenyl sulfone (DDS) was a product obtained from Hanshuo High-tech Materials (Tianjin) Co., Ltd., Tianjin, China. CIP was produced by Chengdu China Magnetic New Technology Development Co., Ltd. (Chengdu, China). The foaming agent, azodicarbonamide (AC), with a decomposition temperature of 215 °C and a gas generation yield of 210 mL/g, was produced by Beijing Loctite chemical products, Beijing, China.

### 2.2. Preparation

Hot melt in situ stirring and dispersion method: The CIP was gradually added to the E51 resin by stirring the paddle until the CIP was completely added. When the mixture was stirred evenly, the curing agent, DDS (40% of the resin content), was added and stirred.

Ball mill dispersion method: E51 and DDS were mixed and stirred evenly according to the ratio of 100:40. And then acetone solvent, whose mass was 2–3 times that of the resin and CIP absorbent, was added. The mixture was poured out after ball milling for 10 h and dried in a vacuum at 90–100 °C for a certain time after the solvent volatilized. When the solvent was volatilized completely and the quality was stable, the ball mill resin/absorbent system was obtained.

The subsequent experiment of foaming was the same for the both dispersion methods as follows: Pre-polymerization was obtained by stirring at 120–140 °C for 10 min, 30 min and 50 min, and then the mixture was cooled to 100–110 °C. The foaming agent, AC (5% of the resin content), was added, and the foaming body was mixed evenly to obtain the pending foam. Then, the pending foam was put into the molding mold at 180 °C for 2–3 h to obtain wave-absorbing foam.

### 2.3. Measurements and Characterization

Electromagnetic parameters of CIP. To explore the intrinsic reasons for the microwave absorption of CIP, 80 wt% CIP was mixed with paraffin and prepared as hollow pipes with the size of 3.04 mm (inner) × 7 mm (outer) × 2 mm (length). The vector network analyzer was used to test in a frequency range of 2~18 GHz with the coaxial line method.

Reflectivity of structural absorbing foam. The reflectivity of the foam was tested with an HP 8722ES vector network analyzer at 2~18 GHz, and the foam size was 200 mm (length) × 200 mm (width) × 10 mm (thickness).

The absorbent/epoxy resin prepared according to different dispersion methods was frozen and quenched. The section was sprayed with gold, and the dispersion of the absorbent was observed using a scanning electron microscope (SEM, Camscan, 3100, London, UK). The average diameters of the bubbles were calculated using image-processing software (Image-Pro Plus 6.0). Cell diameter *D* was computed as the average of the diameters for all of the cells on the SEM micrography and is expressed as the following formula:

(1) $$D=\frac{\sum d_{i}n_{i}}{n_{i}}$$

Compressive properties of the foam were examined according to ASTMD 1621-a standard with specimen dimensions of 50 mm (length) × 50 mm (width) × 26 mm (thickness) and a testing speed of 3 mm/min. The measurements were carried out in an AG-I universal testing machine (Shimadzu, Kyoto, Japan). Five samples were tested in each group, and their average value was calculated.

The foam density of the cylindrical epoxy foams was quantified on cut samples and calculated as follows:

(2) $$\rho_{f}=\frac{m}{V}=\frac{4m}{\pi d^{2}h}$$

where *m* is the mass of the epoxy foam sample. To measure the skeletal density *ρ* of the cured epoxy foam, ~0.5 g ground powder of epoxy foams was subjected to helium displacement pycnometry (AccuPyc l1 1350, Micrometrics, Aachen, Germany).

## 3. Results and Discussion

### 3.1. Absorbent Characteristics

**Figure 2:**
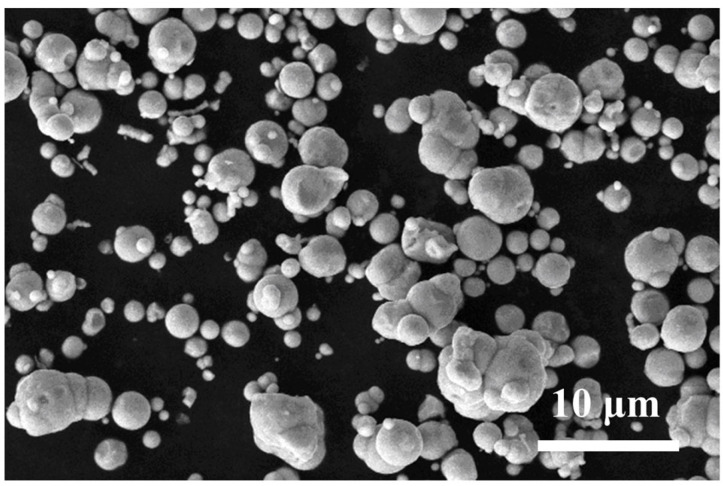
SEM image of CIP.

A Metallic magnetic material is an important magnetic-loss-absorbing material with a high saturation magnetization that is generally more than four times higher than ferrite. The magnetic-loss-absorbing material usually shows a large dielectric constant, high permeability and high electromagnetic loss, which is quite thermally stable with a Curie temperature of above 700 K. The electromagnetic parameters decrease with the increase in frequency, which is conducive to impedance matching and absorption band broadening, making it the main development direction of absorbing materials. CIP is a kind of well-developed magnetic metal powder. Figure 2 shows a SEM image of CIP, and it can be seen that CIP is a relatively regular spherical particle with a particle size of 4~5 μm, which is characteristic of micron-level particles. Its particle structure is like an “onion head” with a smooth surface and low agglomeration [32], so it is easy to disperse evenly in the resin matrix.

**Figure 3:**
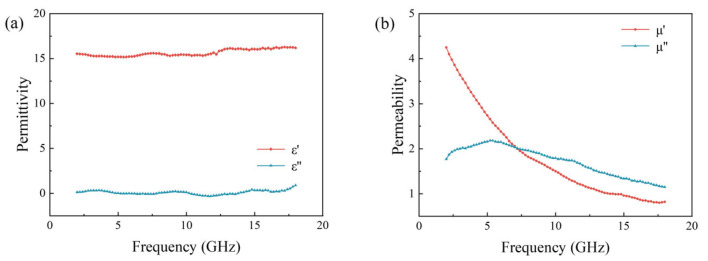
Electromagnetic parameters of CIP (80%) with the matrix of binder. (**a**) Permittivity. (**b**) Permeability.

Figure 3 shows the electromagnetic parameters of CIP. The real part of the dielectric constant of CIP is close to 16, with a small imaginary part close to 0. The real and imaginary parts of the dielectric constant show minimal variation with frequency, indicating that CIP exhibits good microwave absorption performance over a wide frequency range. The real and imaginary parts of the permeability decrease with an increasing frequency, with the real part decreasing at a faster rate. Specifically, at 2 GHz, the values are 4.0 and 1.7, respectively, decreasing to 0.8 and 1.1 at 18 GHz. The CIP shows a good frequency dispersion effect [33]. In an alternating magnetic field of higher frequencies, the change process of both the domain wall displacement and magnetic distance rotation occurs at a finite speed. When the conversion time is less than the relaxation time of the CIP, this means that the magnetization time lags behind the change in the magnetic field. Frequency dispersion effects lead to its electromagnetic parameters. It is very beneficial to achieve broadband absorption for the material with the increase in frequency.

### 3.2. Distribution of Absorbent in Foam

**Figure 4:**
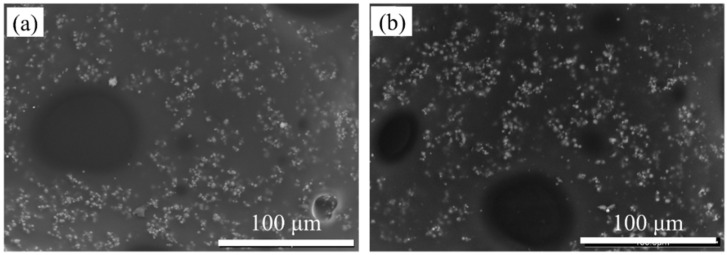
SEM images of CIP/epoxy resin sections with CIP content of 50 wt% prepared by (**a**) hot melt in situ stirring method and (**b**) ball mill dispersion method.

Figure 4 shows the effect of the preparation method on the dispersion of CIP in epoxy resin. It can be seen that the dispersion uniformity of the powder obtained from the ball mill dispersion method is slightly better, but the dispersion of CIP prepared using two different methods is basically in the form of a single particle. This is because the CIP is micron-level and has a smooth surface, so it is not sensitive to the preparation method. Therefore, in the following section, the absorbent is prepared using the ball mill dispersion method.

**Figure 5:**
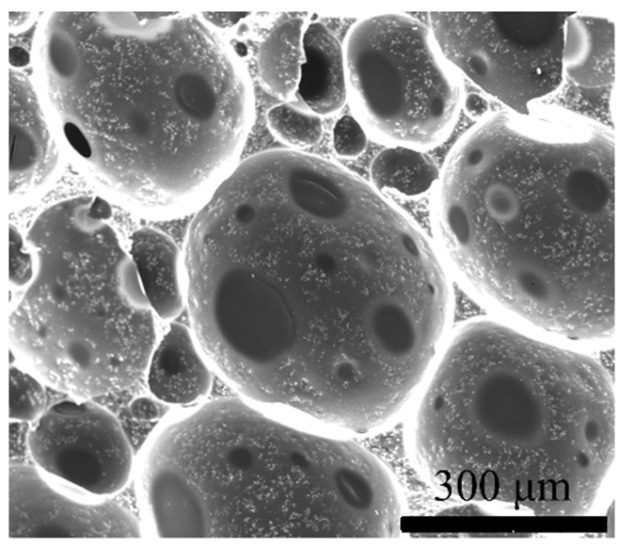
Distribution of CIP in foam with content of 50 wt%.

From the distribution state of CIP in the foam in Figure 5, unlike carbon black, CIP is evenly dispersed in the bubble wall and bubble ridge of the foam. This may be because the CIP is micron-scale and has a smooth surface, and the interaction force between particles is weak. It does not agglomerate easily and is conducive to uniform dispersion.

### 3.3. Effect of Pre-Polymerization Time on Foam Cell Morphology and Properties

The addition of an absorbent will increase the viscosity, which will inevitably affect the subsequent pre-polymerization and foaming processes. Therefore, this section examines the effect of pre-polymerization on the cell structure of the epoxy-based microwave-absorbing foam with the aim of providing a reference for preparing foam materials with a uniform cell structure and good performance.

**Figure 6:**
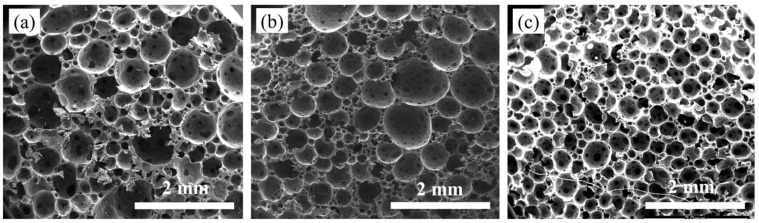
Effect of pre-polymerization time on cell morphology of CIP/epoxy with CIP content of 50 wt%: (**a**) 10 min; (**b**) 30 min; (**c**) 50 min.

**Figure 7:**
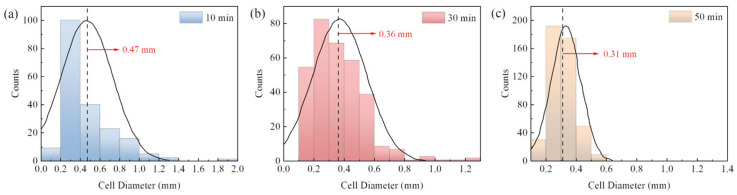
Effect of pre-polymerization time on CIP/epoxy pore size distribution: (**a**) 10 min; (**b**) 30 min; (**c**) 50 min.

There are two main methods for the preparation of chemically foamed materials: one-step and two-step methods. The one-step method involves performing foaming, curing and shaping directly in the resin matrix. The two-step method consists of pre-polymerization and foaming and curing processes. During the pre-polymerization process, the resin system undergoes chain extension, branching and partial crosslinking to achieve a certain degree of curing and viscosity that is sufficient to support the bubbles generated during the foaming and curing processes. The pre-polymerization reaction allows the system to dissipate some of the heat generated, reducing the heat concentration during foaming and curing, preventing scorching and ensuring a more stable and uniform foam structure. The effect of the pre-polymerization time on the morphology and size distribution of CIP/epoxy foam cells is shown in Figure 6 and Figure 7, respectively. The average values are indicated by a dotted line in Figure 7. With the pre-polymerization time extending from 10 min to 50 min, the cell diameter is reduced from 0.47 mm to 0.31 mm. The size distribution follows a typical normal distribution pattern. As the pre-polymerization time increases, the peak shifts towards smaller sizes and the distribution becomes more uniform. This is because, with the prolongation of the pre-polymerization time, the system’s viscosity increases due to the resin crosslinking and curing, matching the gel properties of the resin with the decomposition rate of the foaming agent. As a result, the foam growth stability is improved, and the uniformity is enhanced. With a longer pre-polymerization time, the cell diameter decreases, which is beneficial for the mechanical properties of the foam. Additionally, as the pre-polymerization time increases, the resin viscosity increases, leading to the improved processability of the foam. It is worth noting that further extending the pre-polymerization time would not be beneficial to the process of transforming the resin gel into foam.

**Figure 8:**
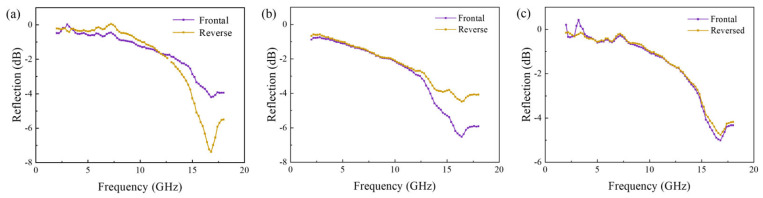
Effect of pre-polymerization time on absorption properties of CIP/epoxy foam with CIP content of 50 wt% and thickness of 10 mm: (**a**) 10 min; (**b**) 30 min; (**c**) 50 min.

The effect of the pre-polymerization time on the absorption performance of CIP/epoxy foam is shown in Figure 8. Interestingly, the absorption performance of the foam differs in the frontal side (the end of the forming direction) and the reverse side (the beginning of the forming direction). The results show that when the pre-polymerization time is short, there is a slight gap between the frontal and reverse absorbing properties, and the frontal absorbing performance is better than the reverse one in the high-frequency band. The reason for this is that the high-density CIP will sink during the foaming process. The CIP would be inhomogenously distributed in a gradient along the foam growth direction, with a lower concentration of CIP on the frontal side and a higher concentration on the reverse side. However, this gap gradually decreases with the extension of the pre-polymerization time. This is because the viscosity of the system increases when the pre-polymerization time is extended, which slows down the sinking of CIP. The reflectivity decreases with the increase in frequency at 2~18 GHz. When the pre-polymerization time is 50 min, the frontal and reverse sides’ absorbing properties are almost the same, and the peak reflectance is 17 GHz, −5 dB.

### 3.4. Effect of CIP Content on Cell Morphology and Properties of Foam

**Figure 9:**
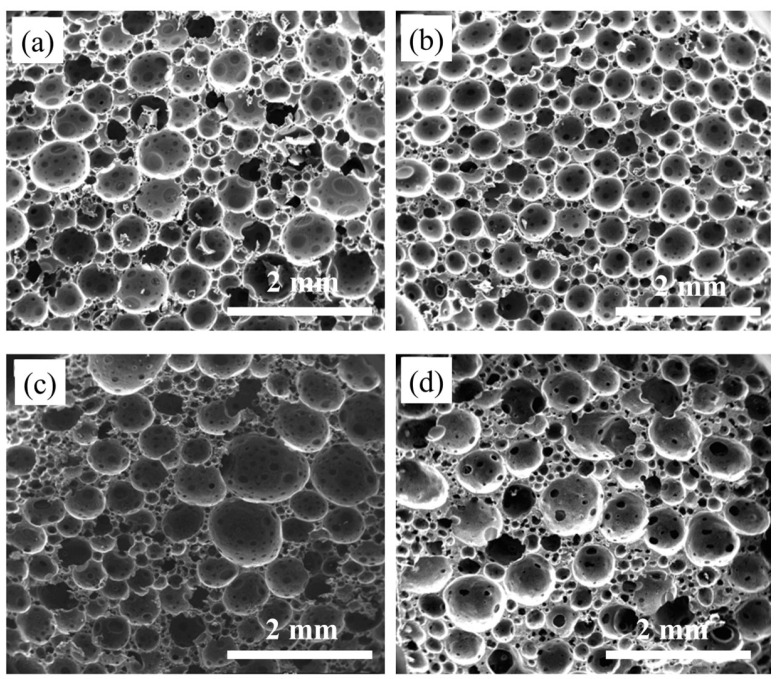
Effect of CIP content on cell structure with pre-polymerization time of 50 min: (**a**) 0 wt%; (**b**) 30 wt%; (**c**) 50 wt%; (**d**) 70 wt%.

**Figure 10:**
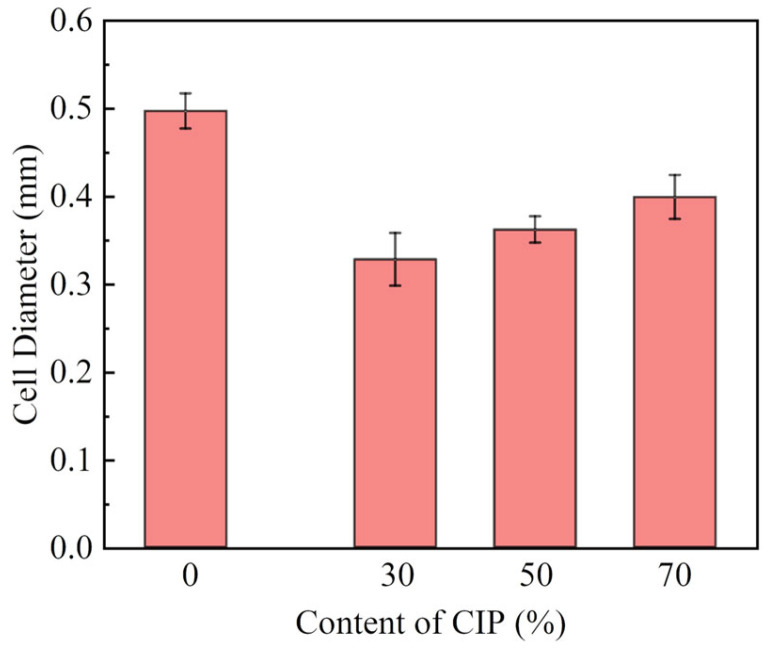
Effect of CIP content on cell diameter: 0 wt%; 30 wt%; 50 wt%; 70 wt%.

Figure 9 shows the effect of the CIP content on the cell morphology, and Figure 10 shows the corresponding cell diameter. The results show that the foam is still a closed-cell structure, that is, the cell structure of the foam changes very little with the addition of CIP. The cell size is smaller than that of blank foam, and the uniformity is also better. The cell diameters are 0.32 mm and 0.40 mm with CIP amounts of 30 wt% and 50 wt%, respectively. With the increase in the CIP content, the cell size gradually increases, and the uniformity of distribution gradually deteriorates because of the large amount of micron-level CIP (>10%). With a further increase in the amount of CIP, the corresponding resin content in the system decreases, and the viscosity increases. Therefore, the difference in the reaction process and degree along the forming direction leads to the aggregation of CIP. Subsequently, weak points are created inside the foam, thus resulting in greater difficulty in the foaming process. Consequently, the sizes of the foam cells increase, and the uniformity becomes poor with further increases in the powder content.

**Figure 11:**
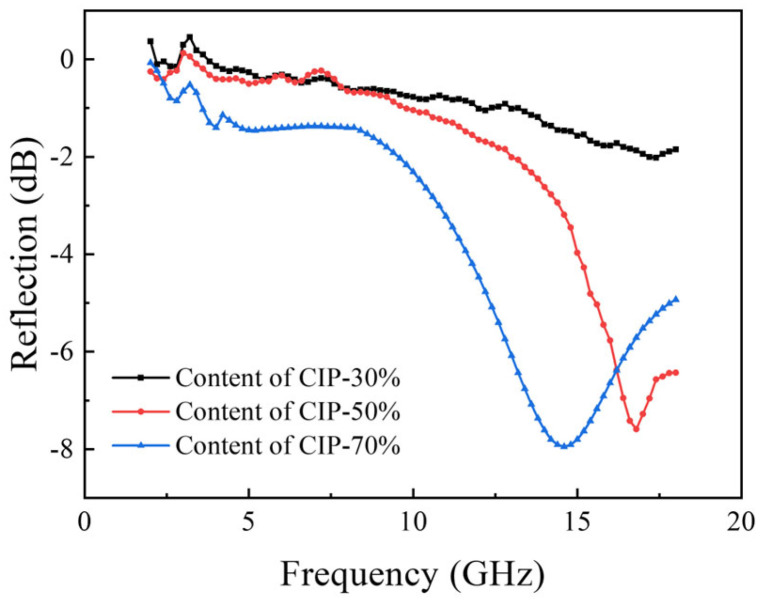
Effect of CIP content on absorption performance with pre-polymerization time of 50 min and thickness of 10 mm.

**Figure 12:**
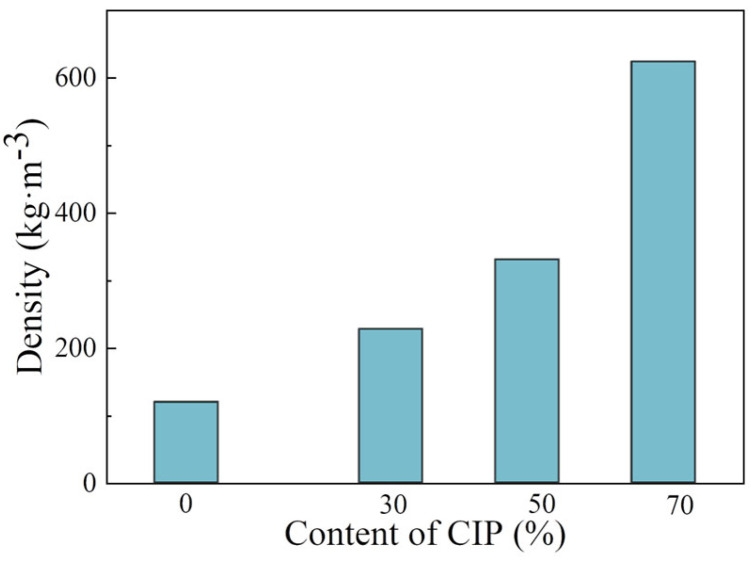
Effect of CIP content on foam density.

Figure 11 shows the effect of the CIP content on the foam absorption performance. The results show that with the increase in the CIP content, the absorption performance gradually increases and the absorption frequency band becomes broader. With the increase in the absorbent content, the electromagnetic parameters (*ε* and *μ*) of the material increase, and then the electrical and magnetic losses of electromagnetic waves increase. Therefore, the absorption peak decreases. With the increase in the amount of absorbent, the absorption peak moves to the low frequency. The reason for this is that the increase in the amount of absorbent is equivalent to the increase in the average dielectric constant *ε*_r_ of the absorbing foam. The increase in the average dielectric constant *ε*_r_ of the foam leads to the increase in the electromagnetic wavelength *λ*_0_ in the free space that it can match, and the corresponding absorption peak frequency is lower. When the CIP content is 50 wt%, the reflectivity of 12~18 GHz is below −4 dB, and the peak reflectance is 14 GHz, −8 dB. When the CIP content is increased, the magnetic pole per unit volume enhances. Increases in the real and imaginary parts of permeability are beneficial to improve the absorption performance. However, due to the high density of CIP, the foam density will increase linearly (230~600 kg/m^3^) with the increase in the CIP content, as shown in Figure 12. To obtain better absorbing properties, a large quantity of CIP is necessary, which will increase the foam density.

Table 1 shows the effect of the CIP content on the compression properties of bismaleimide structure-absorbing foam. The density and foam wall thickness of the foam without CIP are 82.4 kg·m^−3^ and 0.014 mm, which increase linearly with the increase in the CIP content. The foam density increases by about seven times, and the foam wall thickness increases to 0.14 mm with a CIP content of 70 wt%. At the same time, the compression strength and modulus also increase to 3.47 MPa and 270.5 MPa, respectively. The reason for this is that the CIP is evenly distributed in the foam wall and foam ridge of the foam. The modulus of CIP is higher than the epoxy. On the one hand, the addition of CIP leads to the increase in the foam wall thickness, so the ability of the foam to resist deformation is improved. On the other hand, the addition of CIP to the foam compression modulus is greatly increased, which is beneficial to the improvement in the mechanical properties. However, an excessive amount of CIP will lead to a rapid increase in the system viscosity, making it difficult to foam and mold. Therefore, it is important to consider factors such as the material absorption performance, mechanical properties, etc., and select the appropriate CIP content accordingly.

## 4. Conclusions

This study utilized the hot melt in situ stirring method and ball milling dispersion method to prepare structural absorption foam materials with an epoxy resin as the matrix and CIP as the absorber. The following conclusions were drawn:

(1) The dispersion uniformity of the powder obtained using the ball mill dispersion three-roller grinding method is slightly better than that of the powder obtained using the hot melt in situ stirring method. Therefore, the hot melt in situ stirring method was utilized to prepare samples for further investigation. By observing the morphology of the prepared CIP/epoxy foam material, CIP can be uniformly dispersed in the foam material due to its smooth surface and micron-scale structure.
(2) Extending the pre-polymerization times of the resin and CIP is beneficial for reducing the diameters of the foam pores. When the pre-polymerization time is extended from 10 min to 50 min, the foam pore diameter decreases from 0.47 mm to 0.31 mm, resulting in a more uniform distribution of foam pores.
(3) There is a certain gap between the absorption performance of the frontal and reverse sides of the foam when the pre-polymerization time is short. The reflectivity of the two sides almost coincides when the pre-polymerization time is increased to 50 min. The reflectance decreases with the increase in frequency in the range of 2–18 GHz, with a peak value of 17 GHz, −5 dB.
(4) The cell size increases, and the uniformity of distribution becomes poor with a gradually increasing amount of CIP in the composite material. However, increases in the real and imaginary parts of permeability are conducive to improving the absorbing performance. When the CIP content reaches 70 wt%, the reflectivity is lower than −4 dB in the range of 12–18 GHz, and the peak value is −8 dB at 14 GHz. At this time, the wall thickness and density of the foam are significantly improved, and the mechanical properties of the foam material are improved.

## Figures and Tables

**Table 1:** Effect of CIP content on foam compression strength and modulus.

CIP Content (%)	Density (kg·m^−3^)	Foam Wall Thickness (mm)	Compression Strength (MPa)	Compression Modulus (MPa)
0	82.4	0.014	0.58	16.0
30	200.3	0.027	1.13	124.1
50	247.2	0.048	1.32	139.0
70	611.2	0.137	3.47	270.5

## Data Availability

Data are contained within the article.
